# Enhanced photocatalytic degradation of malachite green dye by highly stable visible-light-responsive Fe-based tri-composite photocatalysts

**DOI:** 10.1007/s11356-022-20745-6

**Published:** 2022-05-17

**Authors:** Eman M. Mostafa, Enas Amdeha

**Affiliations:** Egyptian Petroleum Research Institute (EPRI), Nasr City, Cairo, 11727 Egypt

**Keywords:** Environmental pollution, Iron oxide, Malachite green dye (MG), Photocatalytic degradation, Vanadium, Wastewater treatment, Zinc, ZnVFeO_4_

## Abstract

A novel visible-light-sensitive ZnVFeO_4_ photocatalyst has been fabricated by the precipitation method at different pH values for the enhanced photocatalytic degradation of malachite green (MG) dye as a representative pollutant under visible light irradiation at neutral pH conditions. The structure and optical characteristics of the prepared photocatalysts were investigated by XRD, FTIR, N2 adsorption–desorption, TEM, diffuse reflectance spectroscopy (DRS), and photoluminescence (PL) analyses. In addition, the photocatalytic activity of ZnVFeO_4_ photocatalysts superior the efficiency to be more than that of the mono and bi-metal oxides of iron and iron zinc oxides, respectively. The best sample, ZnVFeO_4_ at pH 3, significantly enhances the degradation rate under visible light to be 12.7 × 10^−3^ min^−1^ and can retain a stable photodegradation efficiency of 90.1% after five cycles. The effect of the catalyst dose and the initial dye concentration on the photodegradation process were studied. This promising behavior under visible light may be attributed to the low bandgap and the decreased electron–hole recombination rate of the ZnVFeO_4_ heterostructures. The scavenger experiment confirmed that the hydroxyl radicals induced the MG photodegradation process effectively. Hence, the ZnVFeO_4_ is a reliable visible-light-responsive heterostructure photocatalyst with excellent potential for the photodegradation of organic pollutants in wastewater treatment.

**Supplementary Information:**

The online version contains supplementary material available at 10.1007/s11356-022-20745-6.

## Introduction

In the light of the rapidly increasing population and industrial production, millions of pollutants are being discharged into water bodies causing water scarcity and pollution to become a prominent issue. This problem must be addressed due to the growing damage it poses to water supply security, human health, natural ecosystems, and hence the quality of life. As the water is the origin of life, facing the severe water pollution issue is of great importance and essential to assure clean water to fill the requisites of human life along with other living beings and commercial activities (Nas et al. 2019; Amdeha 2021; Amdeha et al. 2021; Wang and Tang 2021). Manufacturing residues frequently include organic and inorganic pollutants involving heavy metals, synthetic dyes, insecticides, pesticides, pharmaceuticals, etc. These pollutants, notably synthetic organic dyes, regarded as one of the principal pollutants emitted into the environment, must be adequately handled before disposal (Eghbali et al. 2019; Senthil et al. 2020). Dyes are frequently made up of chromophores and auxochromes and have complex molecular structures. The color of the dye is determined by the chromophores, which have an aromatic structure.

Moreover, auxochromes containing –NH_3_, –COOH, –HSO_3_, or –OH groups determine the color intensity and make the molecule water-soluble (Moussavi and Mahmoudi 2009). Synthetic dyes are provided with ecologically more extended durability due to their complex structure and consistent resilience vs. light, temperature, and chemical compounds (Hassani et al. 2020). These dyes in water boost oxygen demand, causing aquatic species to suffer a lot (Gupta et al. 2020). As a result, there is a pressing need to identify more practical, cost-effective, and ecologically friendly technologies for removing dyes before releasing them into water sources to avoid harming aquatic ecosystems (Hassani et al. 2020).

For the treatment of dye effluents, traditional biological, chemical, and physical approaches such as adsorption (Bafana et al. 2011) and chemical precipitation (De Gisi et al. 2016) have been recognized. These processes are affected by high expenses, sludge creation, or secondary pollutant creation, such as dye adsorption on activated carbon material (Bhushan et al. 2020), where the pollutant is just transferred from the liquid phase to the solid phase, producing secondary pollution. Therefore, the decomposition of the dyes into non-toxic products is urgently required and favored. In the degradation process, the aromatic carbon in the dyes decomposes into innocuous CO_2_, whereas N_2_ and S decompose into inorganic ions such as ammonium and sulfate.

To attain the degradation, photocatalytic degradation as one of the advanced oxidation processes (AOPs) is highly recommended and applied (Eghbali et al. 2019; El-Salamony et al. 2021; Deriase et al. 2021; Hassani et al. 2021). AOPs have attracted considerable research interest as effective and economic technologies owing to better oxidizing ability and less secondary pollution (Paździor et al. 2019) due to the highly reactive oxygen species (ROS), e.g., •OH, •O_2_^−^, and ^1^O_2_ produced in the system (Yu et al. 2019; Chen et al. 2022; Hang et al. 2022). When photoactive catalysts like ZnO and TiO_2_ are irradiated with solar/UV light, ROS are created from the oxidation or reduction of different species in the water. Photocatalysis occurs when photons excite photoactive materials. When exposed to sunlight or UV irradiation, photocatalysis generates holes (h^+^) in the valence band (VB) and electrons (e^−^) in the conduction band (CB). To excite (e^−^) from the VB to the CB, the photon’s energy must be higher than the photocatalyst’s bandgap (Bhushan et al. 2020). The reactive radicals that originate from the reaction between the (e^−^) and the oxygen (or the h^+^ and the oxygen) in water react with the organic contaminants in the water, resulting in the formation of non-toxic products such as CO_2_ and H_2_O (Sheydaei et al. 2019; Ghanbari et al. 2019).

Conventional materials, like ZnO, TiO_2_, ZrO_2_, CeO_2_, and SnO_2_ are only active in ultraviolet (UV) light, which accounts for just 4% of the solar spectrum, limiting their usage in simulated or direct solar-light dye degradation over a broad visible range (Banerjee et al. 2014; Nkosi et al. 2014; Ovodok et al. 2018; Natarajan et al. 2018; Yadav et al. 2019; Kannan et al. 2020; Aliabadi et al. 2020; Luque et al. 2021). The advantage of using iron-based photocatalysts is that they can absorb 39% or more of the solar-light composition in visible light and hence have effective degradation efficiencies (Hassani et al. 2018; Madihi-Bidgoli et al. 2021). Hetero-mixed interfaces with more than two metal oxides have shown to be the most successful method for producing visible light photoactive materials. There are various intrinsic structural enhancements at the interface junctions of mixed metal oxides, including greater efficiency in generating, separating, and transporting charge carriers for optimal redox utilization in photocatalytic processes. These photocatalytic processes may be significantly enhanced on the composite oxide surfaces by creating oxygen vacancy defects which can function as electron trapping sites while increasing the reactive substrates’ redox activity adsorption/desorption (Sharma et al. 2021, 2022). Numerous metal orthovanadate (AVO4) (A = metal oxide) depending on BiVO_4_ (Wang et al. 2019), GdVO_4_ (Monsef et al. 2020), CeVO_4_ (Shafiq et al. 2021), InVO_4_ (Shandilya et al. 2019), LaVO_4_ (Wetchakun et al. 2017), and PrVO_4_ (Sajid et al. 2021) semiconductor materials have been successfully fabricated, characterized, and analyzed for photocatalytic structure properties to illustrate the significant photocatalytic improvement in hetero-mixed metal oxides.

The main objective is to enhance the photocatalytic performance by minimizing the bandgap of the prepared photocatalysts to increase the absorption edge toward the visible region to make good use of the solar light as the visible light stands for ~ 43% of the total solar light. Also, as the recombination of the (e^−^) and (h^+^) inhibits the photodegradation process, thus, inhibiting this recombination is of great importance to enhance the degradation efficiency (El-Salamony et al. 2017). Taking these points as motivates, the objective of this article is the improvement and preparation of novel Fe-based photocatalysts for the photocatalytic degradation of the malachite green (MG) dye under visible light (i.e.) with low energy consumption in terms of the degradation efficiencies; and removal (%), rate of reaction (k), and half-life time (t). The photocatalytic degradation process will be under neutral pH conditions to prevent the corrosion that happens when a strongly acidic or alkaline environment is used. Fe-based photocatalysts were successfully synthesized by the precipitation method. The prepared photocatalysts were characterized by different methods for understanding their properties. The effect of the initial dye concentrations and the catalyst dose were studied for maximum removal of MG dye. Through a free radical scavenger experiment, the radical which is responsible for the process was clarified. The recycling experiments were used to study the catalyst stability.

## Experimental

### Materials

In this study, the following chemicals and materials were used. Ammonium hydroxide (NH_4_OH, 28%), ammonium vanadate (NH_4_VO_3_, 99%), ferric chloride anhydrous (FeCl_3_, 99.9%), zinc chloride anhydrous (ZnCl_2_, 99.9%), tert-butyl alcohol ((CH_3_)_3_COH, 98.5%), 1,4-benzoquinone (C_6_H_4_O_2_, 99%), and ammonium oxalate ((NH_4_)_2_C_2_O_4_, 99%) are provided from Sigma-Aldrich. The malachite green (MG) dye (C_23_H_25_N_2_Cl; MW = 364.92 g) is provided from Fluka. All chemicals used in this study were analytical grade and utilized without additional purification.

### Synthesis of pure iron oxide

Pure iron oxide was generated using a simple co-precipitation method in which ammonia solution (NH_4_OH) was added dropwise to anhydrous ferric chloride solution (160 g in distilled H_2_O at 80 °C) while stirring continuously. At pH 9, the reaction mixture was heated for 4 h with rapid mechanical stirring. The created precipitate was allowed overnight at room temperature after complete precipitation, and the resulting solid was centrifuged. The precipitate was washed several times with distilled H_2_O to eliminate ammonium ions before drying at 100 °C. The powder was calcined at 500 °C for 4 h after being crushed in a ball mill (300 rpm for 5 h).

### Synthesis of iron zinc oxide-mixed phases

A solution containing 10 g of anhydrous zinc chloride was dissolved in distilled water. Then, ammonia solution was added dropwise to a vigorously stirred solution containing 40 g of anhydrous ferric chloride in distilled H_2_O at 80 °C to create iron zinc oxide nanomaterial-mixed phase. The reaction mixture was heated for 4 h at pH 9 using a magnetic stirrer with continuous stirring. After complete precipitation, the produced material was centrifuged and washed multiple times with distilled water to eliminate ammonium ions before drying at 100 °C. The powder was calcined at 500 °C for 4 h after being crushed by a ball mill (300 rpm for 5 h).

### Synthesis of iron vanadium oxide-zinc oxide-mixed phases

A solution containing 10 g of ammonium vanadate was dissolved in distilled water. The ammonia solution was added dropwise to a well-stirred solution containing 40 g of anhydrous ferric chloride in distilled water. Then, a solution containing 10 g of anhydrous zinc chloride was dissolved in distilled water at 80 °C. At varying pH values of 3, 9, and 12, the reaction liquid was heated using a magnetic stirrer with continuous stirring for 4 h. After complete precipitation, the precipitated material was centrifuged, then washed multiple times with distilled water to eliminate ammonium ions before drying at 100 °C. The powder was calcined at 500 °C for 4 h after being crushed by a ball mill (at 300 rpm for 5 h). The iron oxide, iron zinc oxide, and iron vanadium oxide-zinc oxide at different pH values are named FO, FZ, FVZ3, FVZ9, and FVZ12, respectively, in which 3, 9, and 12 refer to the used pH value.

### Characterization of the prepared samples

Using a Quantachrome NOVA 3200 automated gas sorption system (USA) at − 196 °C, the surface area of the prepared samples was evaluated using nitrogen adsorption–desorption isotherms. Degassing at 120 °C and 10–5 mm Hg for 3 h was used to prepare the samples. Before and after the activation, the weight of the sample was calculated. The surface area was derived from the adsorption branch using the Brunauer–Emmett–Teller (BET) equation in the relative pressure P/P_o_ range of 0.05–0.35. The Barrett-Joyner-Halenda (BJH) method was used to compute the pore size distribution based on the desorption branches of the nitrogen isotherms. Dynamic light scattering was used to determine particle size distribution (DLS). The Brownian motion of the particles is measured by DLS and related to particle size. A sonic probe was used to combine nanoparticles with solvent. Dynamic light scattering was carried out using Zeta Sized Nano Series, Nano ZS, Malvern Instruments, UK. A Pan Analytical Model X’ Pert Pro with CuK radiation (*λ* = 0.1542 nm), Ni-filter, and general area detector was used to capture X-ray diffraction. A 40-kV accelerating voltage and a 40-mA emission current were employed. The diffraction was recorded using a 0.02 Ǻ step size and a 0.605 2θ scan rate in the 2θ range of 10–80°. Nicolet Is-10 FT-IR spectrophotometer utilizing the KBr method was used to evaluate the prepared samples’ Fourier transform infrared spectroscopy (FTIR): Thermo Fisher Scientific. Because the weight of the sample and the weight of KBr were constantly kept constant, the KBr procedure was carried out in an approximate quantitative manner for all samples. On a JEOL JEM-1230 electron microscope with a 120-kV acceleration voltage, pictures of transmission electron microscopy (TEM) were captured. The samples were made by soaking a carbon-coated copper grid in methanol HPLC for 15 min and coating it with suspended sonicated materials. The UV–vis diffuse reflectance spectroscopy (DRS) study of the produced photocatalysts was performed using a JASCO (Japan) UV-spectrophotometer model V-570, with BaSO4 serving as a standard reflectance reference in the 200–800 nm spectral region. A spectrofluorometer was used to detect photoluminescence (PL) at room temperature (JASCO FP-6500, Japan).

### Photodegradation of malachite green dye

The photodegradation of MG dye assessed the photocatalysts’ performance under both UV and visible light irradiation. The selected dose of the catalyst was dispersed in a known concentration of MG solution under stirring at neutral pH conditions. After adsorption for 1 h, to avoid the interference of the adsorption throughout the photocatalytic processes, a UV (8 W VILBER–LOURMAT; *λ* = 254 nm) or visible light (100 W–TUNGSTEN lamp; *λ* > 400 nm) was turned on for 3 h. Periodically, a sample was collected, centrifuged, and the leftover MG dye concentration was evaluated by a JENWAY–6505 UV–visible spectrophotometer at *λ*_max_ = 615 nm after suitable dilution to determine the efficiency of the reactions via the following equations: Eq. 1, Eq. 2 and Eq. 3. Similar protocol was adopted for parameters study, e.g., light source, photocatalyst dose, dye concentration, pH, and H_2_O_2_ concentration. The reactive oxygen species (ROS) scavenge experiments investigated the degradation mechanism, in which tert-butyl alcohol (BuOH), 1,4-benzoquinone (BQ), and ammonium oxalate (AO) were employed as scavengers for hydroxyl (^•^OH), superoxide (^•^O_2_^−^), and photogenerated (h^+^), respectively (Amdeha and Mohamed 2021). The stability and reusability of the most efficient photocatalyst were evaluated according to the measurements of five consecutive recently prepared 25 ppm MG solutions. The runs were done for 3 h. During the 1st run, MG solution (25 ppm) was poured on the catalyst (1 g/L). After the experiment, under visible light for 3 h at 25 °C, the solution was poured from the vessel, and the residual catalyst was washed with distilled water and then dried. A similar volume of MG solution (25 ppm) was added to the residual catalyst in the second run. The following cycles till the fifth one were repetitive in the same method. The solution temperature during the experiments was kept at 25 ± 1 °C.

Degradation (%)

1 $$\mathrm{D }(\mathrm{\%}) = \frac{\mathrm{Co}-\mathrm{Ct}}{\mathrm{Co}}\times 100$$

The pseudo-first-order kinetic model

2 $$\mathrm{Ln }({\mathrm{C}}_{\mathrm{t}} / {\mathrm{C}}_{\mathrm{o}})=-{\mathrm{k}}_{\mathrm{app}}\mathrm{ t}$$

The half-life time of the dye degradation

3 $${\mathrm{t}}_{1/2}=0.693/{\mathrm{k}}_{\mathrm{app}}$$

where D (%), the degradation efficiency; C_o_, the initial MG concentration; C_t_, the MG concentration at time t; and t_1/2_, the half-life time of the dye degradation.

## Results and discussion

### Characterization of photocatalysts

**Fig. 1:**
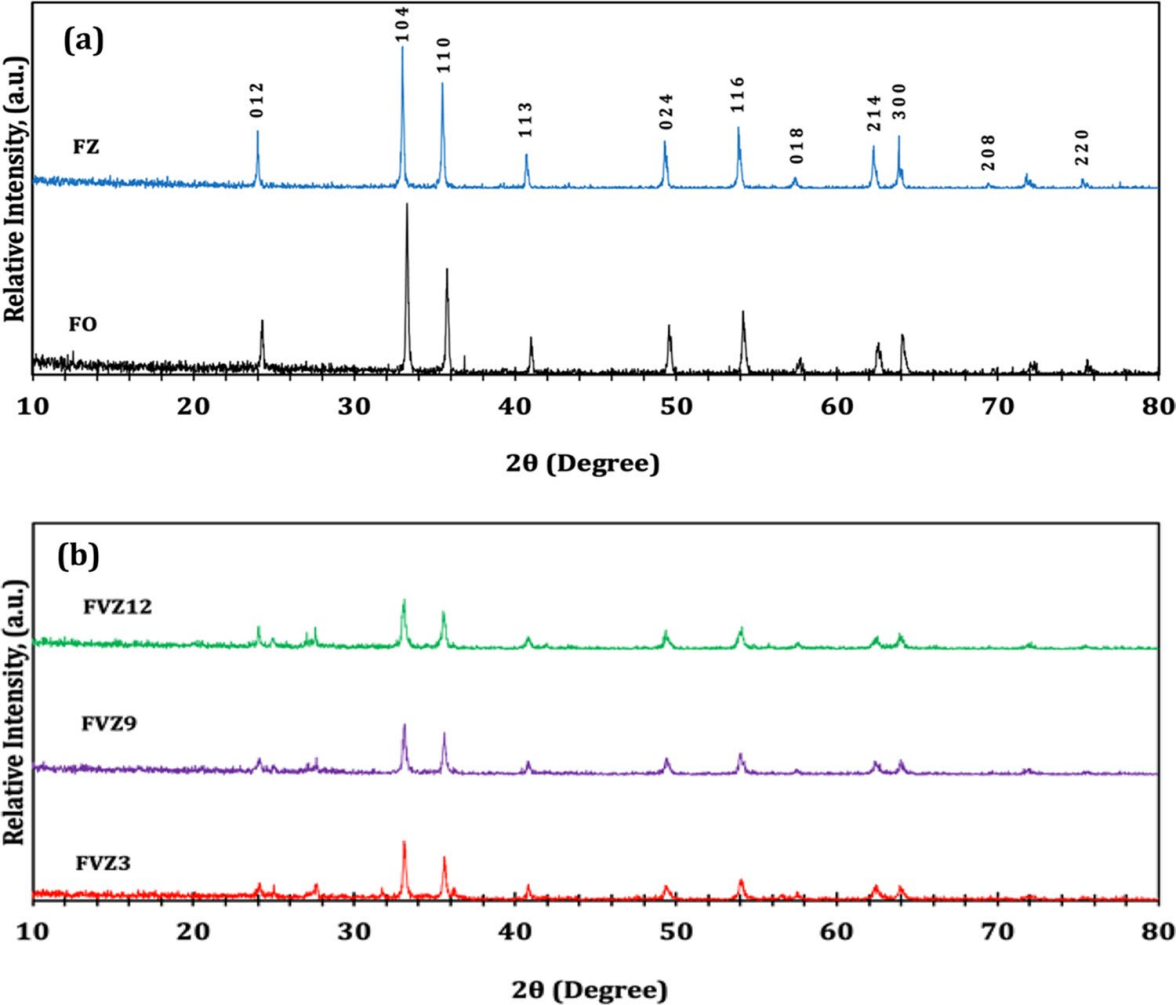
The XRD patterns of (**a**) FO and FZ and (**b**) FVZ photocatalysts at different pH values

The diffraction patterns of the prepared Fe-based photocatalysts, FO, FZ, and various ZnVFeO_4_ at different pH values, were determined by XRD analysis (Fig. 1). XRD patterns of both FO and FZ (Fig. 1(a)) exhibit the characteristic peaks of iron oxide at 23.9°, 32.9°, 35.4°, 40.7°, 49.3°, 53.9°, 57.4°, 62.3°, 63.8°, 69.4°, 71.8°, and 75.3° which correspond to the (0 1 2), (1 0 4), (1 1 0), (1 1 3), (0 2 4), (1 1 6), (0 1 8), (2 1 4), (3 0 0), (2 0 8), (1 0 1 0), and (2 2 0) crystallographic planes of Fe_2_O_3_ (Almeida et al. 2009; Hua and Gengsheng 2009). These twelve peaks are matched to the rhombohedral (hexagonal) structure of hematite (Card no. 04–011-9585). There are no observable peaks for the zinc sample in the XRD pattern due to the low amount of zinc compared to the iron precursor revealing that zinc is embedded into the Fe_2_O_3_ lattice (Lassoued 2021). For FVZ samples (Fig. 1(b)), the peaks for vanadate oxide V_2_O_5_ phase (2θ = 27.64°) and iron vanadium oxide FeVO_4_ phase (2θ = 24.11°) are weak and not well defined due to the dominance of the Fe_2_O_3_ peaks (Elfadly et al. 2013). Moreover, the strong intensity of the Fe_2_O_3_ peaks is relatively decreased. The main peaks at 32.9° and 35.4° exhibited shifting to high 2θ for FVZ samples (e.g., 33.1° and 35.6° for FVZ3) due to the introduction of vanadium into the structure indicating strong interactions of these metals. Additionally, the d-spacing between the atoms became less as the vanadium occupied the interstitial positions leading to changes in the lattice structure (Rahimpour et al. 2020). The Scherer equation, Eq. 4 (Cao et al. 2021), was used to estimate the mean particle size of the produced nanocatalysts (Table 1).

**Table 1:** Specific surface area, cavity structure parameters, and crystalline size of synthesized photocatalysts

Sample	S_BET_ (m^2^/g)	V_Pore_ cm^3^/g	D_pore_ (nm)	Crystalline size (nm)-XRD	d-spacing (Å)
FO	**8.77**	**0.0184**	**42.05**	**48.79**	**2.720**
FZ	**3.25**	**0.0348**	**42.94**	**65.44**	**2.715**
FVZ3	**7.38**	**0.0930**	**50.40**	**30.77**	**2.706**
FVZ9	**5.91**	**0.0652**	**44.12**	**30.16**	**2.705**
FVZ12	**9.43**	**0.1397**	**59.24**	**32.78**	**2.706**

4 $$D= \frac{0.89\lambda }{\beta \mathit{cos}\theta }$$

where D, the mean crystallite size; β, the lengthening of the diffraction line measured at half-maximum intensity; *λ*, the X-ray radiation’s wavelength; and θ, the Bragg angle.

**Fig. 2:**
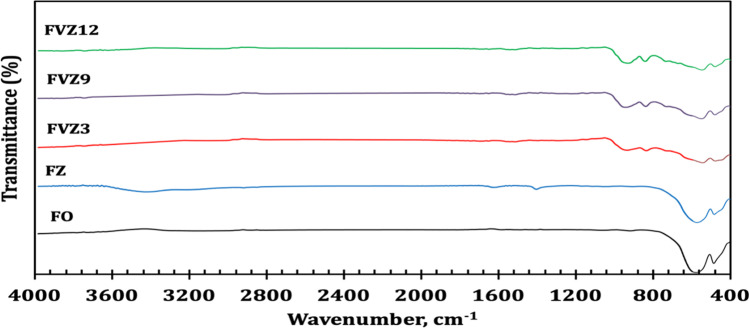
The FTIR patterns of FO, FZ, and FVZ photocatalysts at different pH values

Figure 2 shows the FTIR spectra of FO, FZ, and FVZ nanoparticles and quaternary nanocomposites. The V = O bond’s vibration mode is allocated to the resolved IR band at 953 cm^−1^, whereas the V–O-V bond’s bridging oxygen atoms are attributed to the absorbance peaks between 750 and 850 cm^−1^. The IR bands with significant peaks at 550–700 cm^−1^ are caused by the vibration modes of V–O-Fe and V–O-Fe coupled bridging stretching (Kesavan et al. 2021). The IR bands at 850–1050 cm^−1^are also responsible for the mode of vibration stretch of the short vanadyl (V–O) bonds. The IR band at 894 cm^−1^ is produced by V–O and V–O-V coupled stretching (Sajid et al. 2018; Saravanakumar et al. 2020). The IR bands show the number of modes of vibration peaks at 974–983 cm^−1^, which reduces with higher V_2_O_5_ loadings while producing new larger bands owing to Fe–O and Fe–O-Fe. For FZ, the band at 480 cm^−1^ is associated with Zn–O bonds, whereas for ZnVFeO_4_ nanocatalysts, the band at 470 cm^−1^ is related to ZnVFeO_4_ nanocatalysts. At different pH 3, 9, and 12, a band at 576 cm^−1^ corresponds to F–O stretching vibrations of synthesized FO and FZ nanoparticles and shifts to 550 cm^−1^ ZnVFeO_4_ nanocatalysts.

**Fig. 3:**
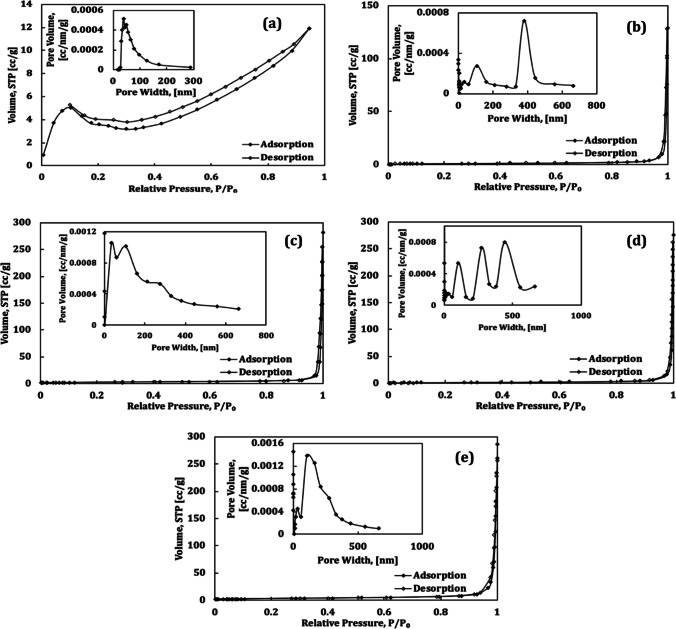
The isotherms and pore size distribution of (**a**) FO, (**b**) FZ, (**c**) FVZ3, (**d**) FVZ9, and (**e**) FVZ12 photocatalysts

The N2 adsorption–desorption isotherms of FO, FZ, and FVZ photocatalyst composites are exhibited in Fig. 3. As can be seen, the samples have type IV adsorption–desorption isotherms with an H3 hysteresis loop, indicative of mesoporous structure based on the IUPAC classification (Malibo et al. 2020; Thaba et al. 2022). The BJH method is used to analyze the pore size distribution (PSD) of the samples in the inset of Fig. 3. The average pore size diameters (D_pore_) and the pore volume (V_pore_) capacity of the Fe-based catalysts vary in the range of 42–59 nm and 0.018–0.139 cm^3^/g, respectively (Table 1). The significant decrease in the surface areas (S_BET_) of Fe-based photocatalysts is due to the introduction of zinc and vanadium oxide in the pores and structure of the catalysts, as confirmed by XRD, resulting in blocking some parts of mesopores and spaces and hence decreasing the surface area (Jiang et al. 2017).

**Fig. 4:**
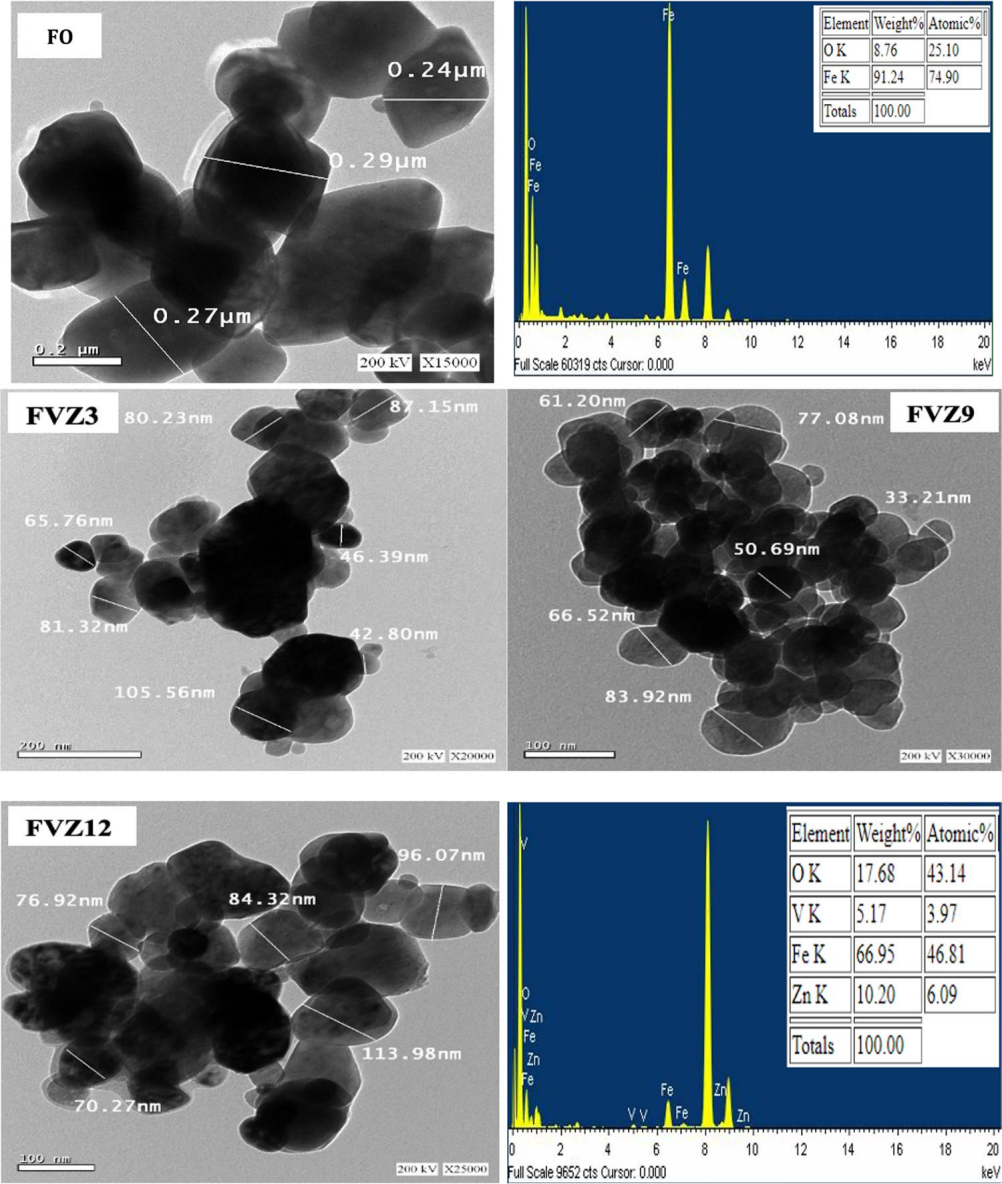
TEM and EDX of FO and FVZ photocatalysts at different pH values

The nanostructure morphology and particle size of Fe_2_O_3_-, FVZ3-, FVZ9-, and FVZ12-based catalysts were investigated using the TEM (Fig. 4). TEM tests revealed that the sample Fe_2_O_3_ had the largest size and hydrodynamic diameter, which indicates how the particles behave in a fluid. The crystalline size increased with increasing pH, reaching 83.92 nm and 113.9 nm for FVZ9 and FVZ12, respectively, due to the incorporation of Zn and V in the structure, as confirmed by XRD. The Fe, V, and Zn components are evenly distributed in the ZnVFeO_4_ catalysts. (Fe = 66.95 wt %); (V = 5.17 wt %); and (Zn = 10.20 wt %) were the preliminary metal levels in the FVZ catalysts.

The DRS and PL spectra of the Fe-based photocatalysts were determined to study their optical characteristics. The DRS was done to assess the optical properties and the bandgap of the Fe-based photocatalysts. The results are shown in Fig. S1. All three tri-composites, FVZ, exhibit a strong absorption band in both UV and visible regions, implying that these photocatalysts can be excited by an extensive range of light (Hang et al. 2022), and have excellent performance toward visible light response, indicating the photocatalytic ability under visible light. The optical band gap was calculated via the following equations: Eq. 5, Eq. 6 (Yu et al. 2018; Gupta et al. 2020):

5 $${(\mathrm{\alpha h\upsilon })}^{1/2}=\mathrm{A}(\mathrm{h\upsilon }-{\mathrm{E}}_{\mathrm{g}})$$

6 $$\mathrm{\alpha }={(1-\mathrm{R})}^{2}/2\mathrm{R}$$

E_g_, the bandgap energy(eV); h, Planck’s constant; ν, light frequency; hν, the photon energy (eV); A, a proportional constant; α, the absorption coefficient; R, reflectance.

The bandgap energies of FVZ3, FVZ9, and FVZ12 are 1.90, 1.91, and 1.96 eV calculated from the Tauc method (Fig. S1). The inclusion of metal energy levels may have reduced the energy gap of the electronic transition, resulting in a narrow bandgap for FVZ (Kang et al. 2015). Besides, the excellent visible-light absorption could enhance the photocatalytic performance of the FVZ3 sample as it has the smallest bandgap (Manchala et al. 2021).

**Fig. 5:**
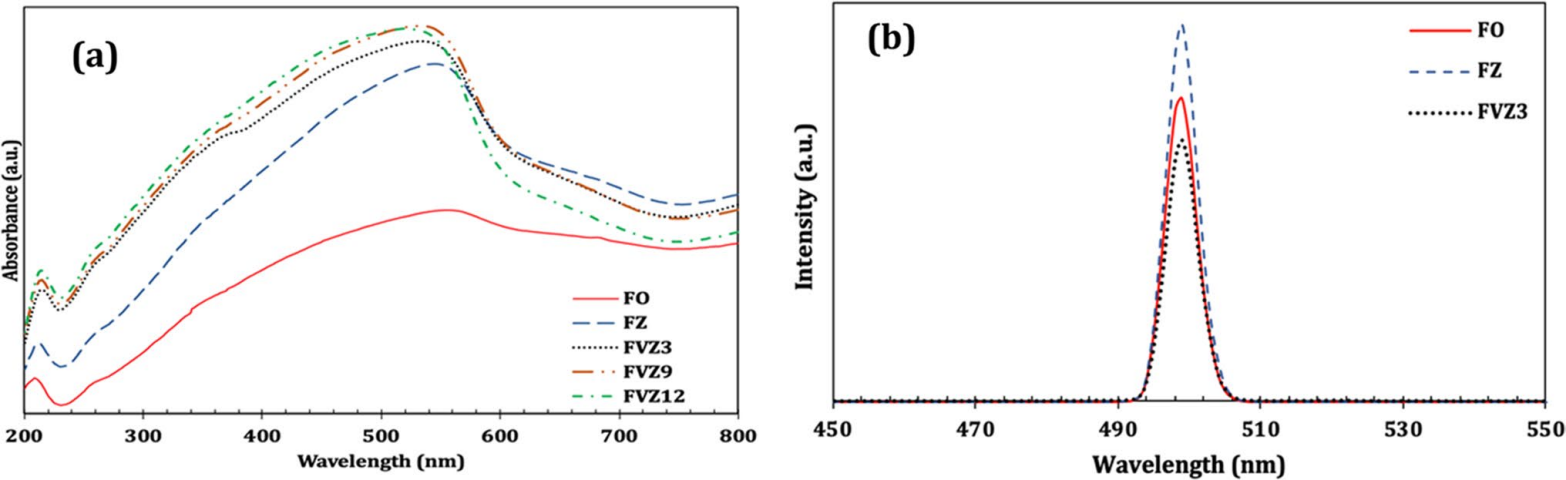
UV–vis DRS spectra (**a**) and PL spectra under an excitation wavelength (*λ* = 254 nm) (**b**) of the prepared photocatalysts

The separation of the photoinduced (e^−^)–(h^+^) pairs is crucial from the photocatalytic point of view. The behavior of these photo-generated charge carriers can be revealed through the PL measurements. In general, a high recombination rate of photogenerated electrons and holes suggests a high fluorescence intensity in PL spectra, whereas a low recombination rate can improve photocatalytic performance (Liu et al. 2022). The PL spectra of the Fe-based nanocomposites (Fig. 5) exhibit a main peak at 499 nm, which is referred to the recombination of excited (e^−^) and (h^+^) in the conduction and valence bands, respectively. This peak is attributed to the visible light emission caused by defects such as oxygen vacancies or Zn interstitial (Thein et al. 2016). A significant intensity variation is due to the different samples’ compositions. A decrease in the intensity of the FVZ3 is owing to the weak fluorescence response as compared to the FO and FZ undoubtedly shows that the recombination of photogenerated (e^−^)–(h^+^) is minimized in the tri-composite (Liu et al. 2022). This indicates that the FVZ3 composite can display more significant photocatalytic activity to the dye degradation than the other photocatalysts. The narrow bandgap of FVZ3 at 1.90 eV may encourage lattice defects under UV–visible light irradiation, which can act as an exciton center that hinders the recombination to enhance its photoactivity (Li et al. 2011).

### The photocatalytic activity of the visible-light-responsive Fe-based photocatalysts

**Fig. 6:**
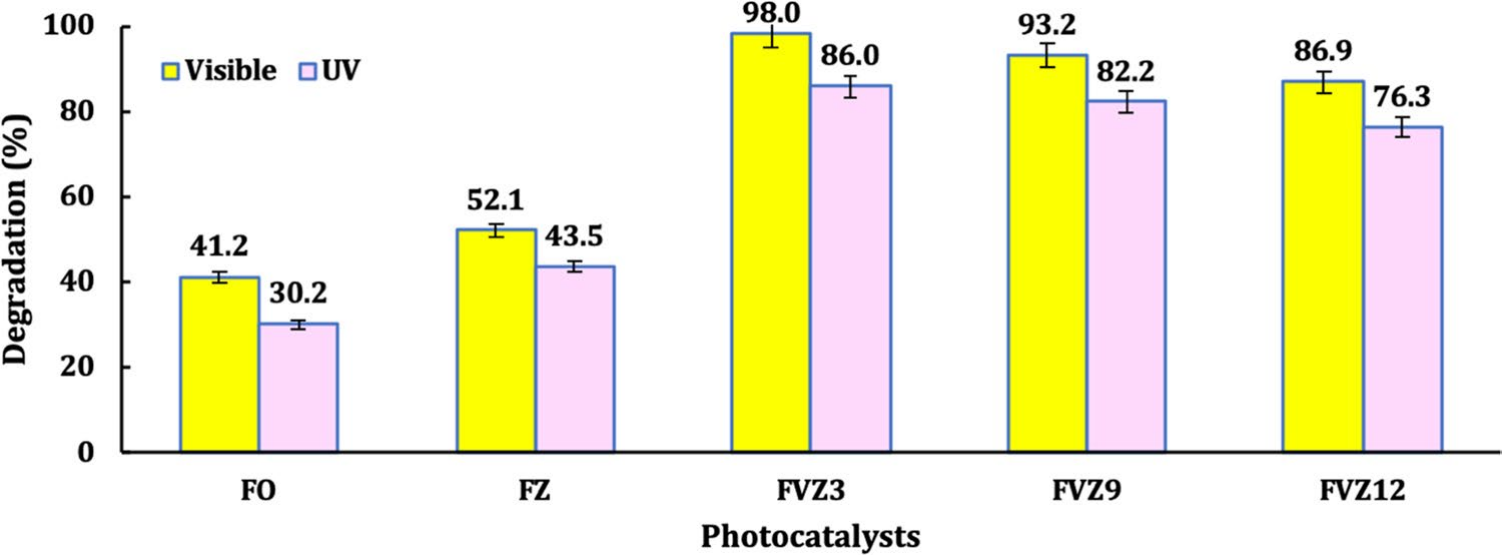
Effect of different photocatalysts on the MG photocatalytic degradation under both UV and visible light irradiation (photocatalyst dose = 1 g/L, [MG] = 25 ppm)

The activity of the visible-light-sensitive Fe-based photocatalysts was evaluated using malachite green (MG), one of the industrial dyes’ effluents, as a target organic compound model under visible light irradiation. The performance of the Fe-based photocatalysts, i.e., FO, FZ, FVZ3, FVZ9, and FVZ12, was found unsatisfactory in the absence of light, i.e., adsorption, with only 3–4% of dye was removed after 60 min without light irradiation. Also, in the absence of the photocatalyst, i.e., direct photolysis, less than 3% of dye was photodegraded under UV and visible light irradiation. It can be concluded that the MG removal efficiency is relatively poor under photolysis and adsorption. Consequently, a high-performance photocatalyst and suitable light source are required conditions for effective MG degradation (Du et al. 2020). The results show that the process can reach a saturation adsorption state within 60 min prior to the light irradiation and has almost no effect on the photodegradation performance and the rate. Figure 6 shows the MG degradation (%) after 180 min for the catalysts under UV and visible light irradiation. From this figure, the FVZ3 sample provides the highest MG photodegradation efficiency of 98% under visible light, while the photocatalytic performance of the same sample offers only 86% under UV light. This is due to the low band-gap energy of the FVZ3 photocatalyst, as mentioned in the optical properties. Also, this behavior is coincident with the previous study (El-Salamony et al. 2017) in which, under UV light, the radical recombination exceeds the separation, while under visible light, the electron–hole generation is prevalent, and the recombination is poor. This behavior could prove the catalyst activity under visible light and subsequently under sunlight, in which no energy consumption is required, which is a good point from an economic and energy-saving point of view. Therefore, visible light is nominated for further experiments due to the outstanding photocatalytic degradation efficiency.

The photocatalytic degradation of pollutants by photocatalysts generally follows the pseudo-first-order-kinetic model (Chiang and Doong 2015). Table 2 presents the reaction rate constant k, the half-life time t_1/2_, and the degradation (%) acquired by Eq. 1, Eq. 2, and Eq. 3. From Table 2, the calculated k values were 3.8 × 10^−3^ min^−1^, 5.5 × 10^−3^ min^−1^, 12.7 × 10^−3^ min^−1^, 11 × 10^−3^ min^−1^, and 9.5 × 10^−3^ min^−1^, for FO, FZ, FVZ3, FVZ9, and FVZ12, respectively. Noteworthy, the FVZ3 reveals the highest rate constant, k of 12.7 × 10^−3^ min^−1^, approximately 3.3 times higher than the FO sample. This result shows that the MG photodegradation rate follows the order FVZ3 > FVZ9 > FVZ12 > FZ > FO. FO and FZ show medium photocatalytic activity for MG degradation under visible light, possibly due to the rapid recombination of the photogenerated carriers (Ahmad et al. 2019). While FVZ3 exhibits higher activity than other photocatalysts, this agrees well with the optical data derived from the PL spectra. It is also worth noting that the photocatalytic degradation of organic compounds is a surface-mediated reaction (Han et al. 2016), with dye adsorption onto photocatalyst surfaces being the first step. Cationic MG dye can simply be adsorbed onto the negatively charged catalysts and FVZ3 exhibits excellent photocatalytic performance and rate compared to the other prepared photocatalysts due to its relatively high pore volume.

**Table 2:** Determination of the degradation %, pseudo-first-order rate constant, k (min^−1^), half-life time, t_1/2_ (min), and determination coefficient (*R*^2^), under visible light irradiation (dose = 1 g/L, conc. = 25 ppm)

Catalyst	Degradation %	k (min^−1^)	t_1/2_ (min)	*R*^2^
FO	41.2	0.0038	182	0.97
FZ	52.1	0.0055	126	0.97
FVZ3	98.0	0.0127	55	0.99
FVZ9	93.2	0.011	63	0.99
FVZ12	86.9	0.0095	73	0.99

### Factors affecting the photocatalytic degradation of MG dye

**Fig. 7:**
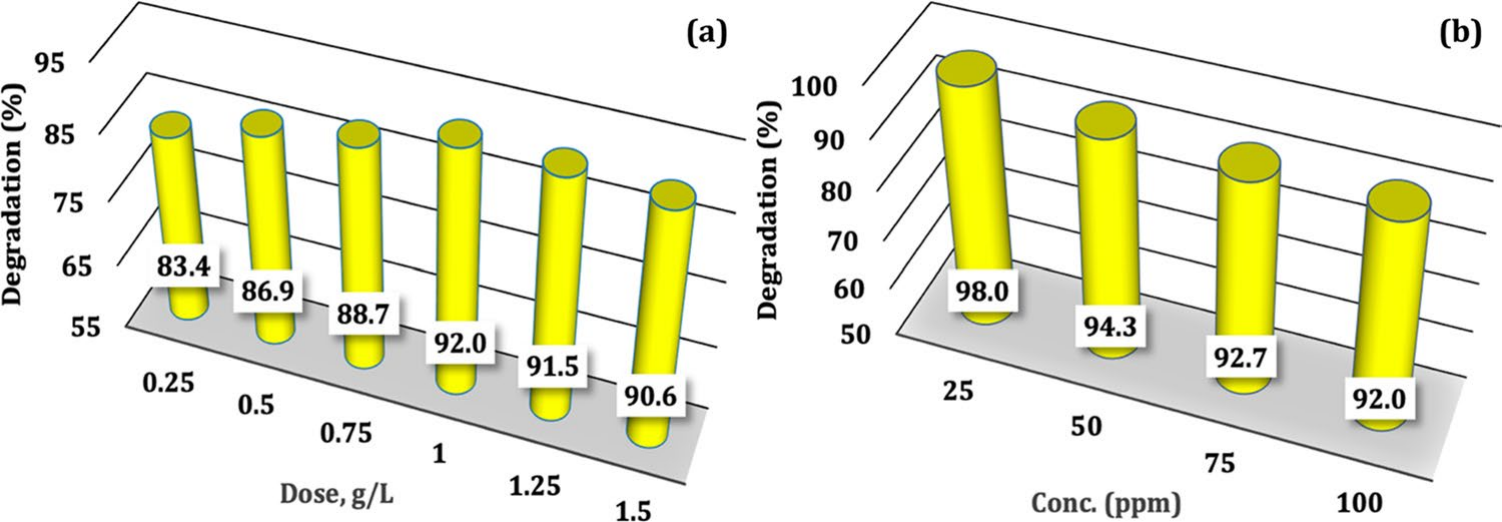
**a** Effect of photocatalyst dosage ([MG] = 100 ppm) and (**b**) effect of MG concentration ([FVZ3] = 1 g/L) on the photocatalytic degradation under visible light irradiation

To determine the best conditions for MG photodegradation, reasonable runs are conducted. The impact of FVZ3 dosage on the photodegradation of the MG dye is displayed in Fig. 7(a). As the dose increases from 0.25 to 1 g/L, the MG degradation enhanced from 83.4 up to 92.0% for the degradation of 100 ppm MG. As the quantity of FVZ3 composite photocatalyst increased, the photoactive sites also increased, leading to an increase in the degradation efficiency. The increased catalyst dose enables a larger surface area for catalyst interaction and a higher concentration of free radicals per mL of the MG solution, resulting in a higher dye removal percentage (Xu and Wang 2011; Bhushan et al. 2020). When the dose was increased to 1.25 g/L and 1.5 g/L, the degradation efficiency dropped to 91.5% and 90.6%, respectively. Excess catalyst in the solution can prevent visible light from reaching the reaction suspension, resulting in a loss of accessible radiation and, as a result, low photoactive sites (Li et al. 2019). As a result, the best dosage for MG photodegradation using FVZ3 was 1 g/L.

The photodegradation of different MG initial concentrations was studied under visible light irradiation for all Fe-based photocatalysts with 1 g/L as a catalyst dose (Fig. 7(b)). The efficiency of MG photodegradation reduces as the starting dye concentration rises from 25 to 100 ppm. From Fig. 7(b), after 180 min of visible light irradiation, a nearly complete photodegradation (~ 98%) of 25 ppm MG by 1 g/L of FVZ3 is seen. When the starting concentration is increased to 100 ppm, only 92% of the MG is photodegraded. The inhibitory impact of the adsorbed MG molecules on light penetration could explain the decrease in MG photodegradation at high starting concentrations. It is worth noting that MG is a green dye, and thus, increasing its concentration would cause it to absorb more light. As a result, increasing the initial MG concentration reduces photon penetration to the photocatalyst surface, lowering the MG photocatalytic degradation efficiency (Pitchaimuthu et al. 2014). Another possible explanation for the lower efficiency is the photocatalyst’s restricted number of active sites.

### Reusability of the catalyst

**Fig. 8:**
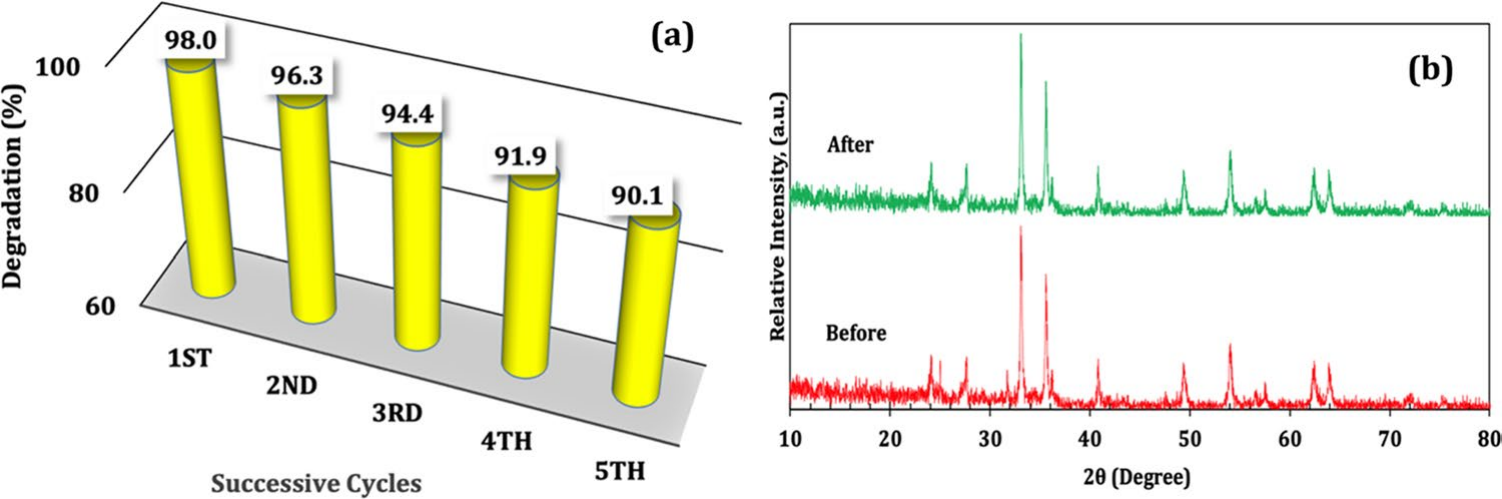
**a** Effect of FVZ3 recycling on the MG photocatalytic degradation ([FVZ3] = 1 g/L and [MG] = 25 ppm) and (**b**) XRD patterns of FVZ3 before and after five experimental runs

The reusability of the FVZ3 photocatalyst is of supreme significance from a financial standpoint because of the cost related to the catalyst preparation. The reusability was investigated by successive reusability tests, in which the photodegradation efficiencies of the FVZ3 sample in five continuous cycles were tested. Figure 8(a) displays the reusability test of the 25 ppm MG with 1 g/L of catalyst dose under visible light irradiation. After the third cycle, FVZ3 still displays an excellent visible-light responsive activity with a high MG degradation of about 94.4%. The third cycle efficiency is decreased only by 3.6% from the first one, which may be caused by the accumulation of intermediates after the MG photocatalytic degradation (Nguyen and Doong 2016).

Furthermore, the structure of the used FVZ3 was characterized using XRD (Fig. 8(b)). Compared with the freshly prepared sample, the recovered catalyst shows no significant structural changes in the XRD of the FVZ3 composite, which indicates its superior stability. The multiple catalyst reusability makes it a good choice for photodegradation of the pollutants existing in the water.

### Photocatalytic reaction mechanism (scavengers test)

**Fig. 9:**
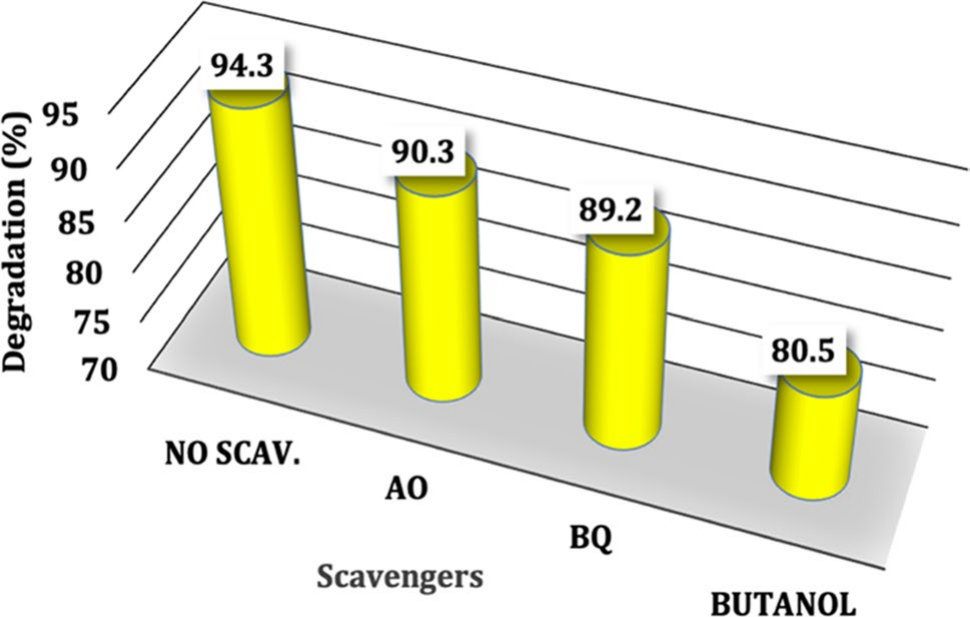
MG photocatalytic degradation by FVZ3 composite under visible light irradiation with/without scavengers (]MG[= 50 ppm,]FVZ3[= 1 g/L)

Reactive species have a crucial role in the photodegradation process of the contaminants. Superoxide radical anions (^•^O_2_^–^), hydroxyl radicals (^•^OH), and photoinduced holes (h^+^) are among the reactive species. The decrease of the photodegradation activity reveals the vital role of the active radicals. The influence of the active species follows the order ^•^OH > ^•^O_2_^–^ > h^+^ agreeing to the results in Fig. 9. The MG degradation % decreased from 94.3 to 90.3%, 89.2%, and 80.5% after adding AO, BQ, and BuOH scavengers, respectively. Agreeing to the obtained results, the introduction of t-butyl alcohol significantly could reduce the MG degradation efficiency, indicating that the ⋅OH radicals are the principal active species. Another contributed ROS is ^•^O_2_^–^ because the presence of BQ significantly reduces the MG degradation efficiency. In contrast, a slight decrease in the efficiency was detected in the presence of the AO, suggesting that h^+^ has a limited contribution to the MG photodegradation (Chen et al. 2022).

Based on the previous analysis, a probable mechanism for MG degradation was proposed, in which the ⋅OH may directly oxidize MG dye. The bandgap of the composite catalyst has been lowered, and the visible light response has been improved. As a result, more electrons will be excited from the valence band into the conduction band of FVZ3 and produce photo-generated electron–hole pairs during visible light irradiation.

In addition, the low recombination rate of photo-generated electron–hole pairs for FVZ3 enhances the photocatalytic degradation activity. The photo-generated (e^−^)–(h^+^) pairs split quickly and travel to the photocatalyst’s surface. The effectual separation of the photo-generated (e^−^)–(h^+^) pairs results in more electrons participating in the continuous degradation reaction (Eq. 7). The electrons react with O_2_ to produce ^•^O_2_^–^ and ^•^OH (Eq. 8 and Eq. 9) (Chen et al. 2022); these radicals, in turn, degrade the MG molecule.

7 $$FVZ3 + h\upsilon \to {e}^{-}+{h}^{+}$$

8 $${O}_{2}+{e}^{-} \to \bullet {O}_{{2}^{-}}$$

9 $$2 \bullet {\mathrm{O}}_{{2}^{-}}+{2\mathrm{H}}^{+}\to 2 \bullet \mathrm{OH}+{\mathrm{O}}_{2}$$

10 $$\bullet \mathrm{OH}/\bullet {\mathrm{O}}_{{2}^{-}}+\mathrm{MG}\to \mathrm{degraded products}$$

### Comparison of FVZ3 photocatalyst efficiency with literature

The FVZ3 composite was compared with some previously reported photocatalysts regarding initial MG concentration, photocatalyst dose, irradiation source, time, and degradation (%). From Table 3, it can be concluded that the current study provides a more efficient photocatalyst that can degrade high MG concentrations up to 100 ppm with a reasonable dose of 1 g/L under visible light irradiation. From an economic and energy-saving point of view, these valued data suggest that FVZ3 would be a promising photocatalyst for the photocatalytic degradation of dyes.

**Table 3:** Photocatalytic degradation of MG dye by various photocatalysts in aqueous solution in the literature

Catalyst	Initial MG concentration (ppm)	Catalyst dosage (g/L)	Light source	Irradiation time (min)	Degradation (%)	Ref
Chitosan/Ce–ZnO	5	0.2	Visible	90	100	(Saad et al. 2020)
^**1**^Fe@(Alg-CMC)	10	2	UV	30	98.8	(Karadeniz et al. 2022)
10%rGO/FeVO_4_	10	1	Sunlight	120	100	(Alsulami et al. 2021)
0.2% La-ZnO/SiO_2_	15	0.3	Visible	140	96.1	(Wang et al. 2021)
ZnO-La_2_CuO_4_	25	0.1	Visible	120	91	(Yulizar et al. 2020)
ZnO/CNT	30	0.1	Visible	60	79	(Arsalani et al. 2020)
Ag/ZnO/g-C_3_N_4_	40	1.6	Visible	60	91	(Mohanty et al. 2021)
Fe–N-C/CN_*x*_-700	50	0.2	Visible	140	92.1	(Huang et al. 2022)
C_ZnO_-dots	50	0.05	Visible	60	94.8	(Sekar and Yadav 2021)
^**2**^XG/AA@ZnO BNC	70	7	Xenon	46	99.45	(Bassi et al. 2022)
FVZ3	100	1	Visible	180	92	This study
FVZ3	25	1	Visible	180	98	This study

^1^Fe(III) ion cross-linked alginate-carboxymethyl cellulose composite beads

^2^Xanthan gum/agar@ZnO

## Conclusion

In this study, a novel ZnVFeO_4_ has been successfully fabricated at different pH by precipitation method. The enhanced photocatalytic degradation of MG dye as a model pollutant at neutral pH conditions under visible light irradiation has been investigated. The XRD and FTIR analyses confirmed the presence of Fe, Zn, and V in the ZnVFeO_4_ samples with crystalline size in the range of 30–65 nm. The optical properties indicate that all the prepared Fe-based photocatalysts absorbed effectively in the UV–Vis region and have a low bandgap. The calculated bandgap from DRS was in the range of 1.9–1.96 eV for ZnVFeO_4_ at different pH values. Compared to the FO sample, the low bandgap and the decreased photoluminescence intensity at around 488 nm of the FVZ3 accelerated the rate and photodegradation efficiency of MG dye under visible light irradiation. The photodegradation rate of MG dye by the prepared photocatalysts follows the order FVZ3 (12.7 × 10^−3^ min^−1^) > FVZ9 (11 × 10^−3^ min^−1^) > FVZ12 (9.5 × 10^−3^ min^−1^) > FZ (5.5 × 10^−3^ min^−1^) > FO (3.8 × 10^−3^ min^−1^). The effect of the catalyst dosage and the initial dye concentration on the photocatalytic performance were elucidated. The optimum catalyst dose is 1 g/L to attain a high degradation efficiency with a low cost. Furthermore, cycling experiments for the fabricated photocatalyst demonstrated remarkable stability and recyclability of FVZ3 with high photocatalytic efficiency (> 90%) even after five cycles of use under visible light irradiation. The dye degradation probable mechanism was driven by studying the process efficiency under the existence of the scavengers, in which the contribution of the active species follows the order ^•^OH > ^•^O_2_^–^ > h^+^, confirming the primary role of ^•^OH and ^•^O_2_^–^. According to these findings, these photocatalysts have the potential to be employed without changing the pH for the cost-effective treatment of dye-contaminated wastewater when exposed to visible light.

## Supplementary Information

Below is the link to the electronic supplementary material.

Supplementary file1 (DOCX 108 KB)

## Data Availability

All obtained data during this work are included in this manuscript.
