# Supplementary material for: Enhanced photocatalytic degradation of malachite green dye by highly stable visible-light-responsive Fe-based tri-composite photocatalysts
Source: Environ Sci Pollut Res Int. 2022 May 17;29(46):69861–74. doi: 10.1007/s11356-022-20745-6 (PMC9512746; doi:10.1007/s11356-022-20745-6)
Supplement: Supplementary file 1 — Supplementary file1 (DOCX 108 KB) [file 11356_2022_20745_MOESM1_ESM.docx]

Supplementary Information

**Enhanced photocatalytic degradation of Malachite Green Dye by highly stable visible-light-responsive Fe-based tri-composites photocatalysts**

Eman M. Mostafa and Enas Amdeha*

Egyptian Petroleum Research Institute (EPRI), Nasr City, Cairo 11727, Egypt.

[*enas_amdeha@yahoo.com](mailto:*enas_amdeha@yahoo.com), +201002771065

|  |
| --- |
| **Fig. S1.** The Tauc plot of FVZ3, FVZ9, and FVZ12 photocatalysts. |
